# Employment of chia gum and tannic acid as natural products in immobilization of horseradish peroxidase for the removal of azo dye

**DOI:** 10.1186/s12896-025-01058-1

**Published:** 2025-12-01

**Authors:** Azza M. Abdel-Aty, Amal Z. Barakat, Hala A. Salah, Saleh A. Mohamed

**Affiliations:** Molecular Biology Department, National Research Centre, Dokki, Cairo, Egypt

**Keywords:** Tannic acid, Chia gum, Horseradish peroxidase, Immobilization

## Abstract

**Background:**

The numerous hydroxyl groups in tannic acid (TA) and its tendency to form hydrogen bonds with proteins and carbohydrates are key factors contributing to its utility. TA-based enzyme immobilization supports provide flexible platforms for enzyme immobilization. chia gum (CG) can also trap significant amounts of water between its chains, preventing water loss from the gum and inhibiting enzyme leaching.

**Results:**

In this study, TA, a natural linker molecule, was used to immobilize horseradish peroxidase (HRP) on chia gum (CG), serving as the natural support. On the created TA-CG support, HRP was successfully immobilized, with the maximum immobilization recovery (80%) observed at pH 7.0, 9 mg TA, and 20 U of the enzyme. Using a scanning electron microscope (SEM) and Fourier transform infrared (FTIR) technology, the chemical functional groups and morphological characteristics of the synthesized CG-TA-HRP were illustrated. After ten reuses, CG-TA-HRP demonstrated good reusability, with 65% retention. By comparing CG-TA-HRP to soluble HRP, the former exhibited better thermal stability up to 40 °C, a higher temperature optimum at 50 °C, and a pH optimum at 6.0. According to the K_m_ data, CG-TA-HRP exhibited a lower affinity for hydrogen peroxide (H_2_O_2_) and guaiacol and a higher oxidizing affinity to certain phenolic substrates. The prepared CG-TA-HRP demonstrated greater resistance to heavy metals, isopropanol, urea, and Triton X-100. During the 6-hour incubation period, soluble HRP removed 44% of the methyl orange, while immobilized HRP decolorized 78% of the dye.

**Conclusions:**

The TA-linker molecule and chia gum are considered environmentally safe components in the production of CG-TA-HRP, making this an easy and eco-friendly method for enzyme immobilization.

## Introduction

Peroxidases, associated with electron oxidation, oxidize a wide range of aromatic compounds [1–3]. Peroxidases typically convert oxidized aromatic molecules into less water-soluble metabolites. Peroxidases are used in the bioremediation of phenol derivatives and dye decolorization [4, 5]. Wastewater from various industries, including the paper and textile industries, contains azo dyes. According to several studies, azo dyes are widely considered to be hazardous substances that may lead to cancer [6, 7]. Therefore, azo dyes must be eliminated before wastewater can be discharged into agricultural land [8]. Azo dyes typically oxidize by peroxidases to produce less hazardous chemicals [9, 10].

Free enzymes have several limitations that may hinder their industrial use. Factors such as enzyme activity, selectivity, and specificity towards the targeted industrial substrate, as well as enzyme purity and stability under industrial conditions, are crucial in determining whether the use of enzymes in industrial processes is viable [11–13]. Free enzymes are difficult to remove from reaction media, resulting in the loss of high-activity enzymes. This leads to the need for frequent enzyme replenishment, reducing catalytic activity and increasing process costs [14–15].

Enzyme immobilization enhances enzyme stability, protects the product from enzymatic contamination, and permits continuous processing, all of which boost process economics. The study of enzyme immobilization has been conducted using a variety of solid materials, both organic and inorganic [16]. As a result, immobilized enzymes are widely used in industries such as food and pharmaceuticals, and in bio-separators and biosensors [17]. Covalent binding of enzymes to water-insoluble carriers is considered the most effective immobilization method for enhancing enzyme stability, reusability, and recovery, compared to other techniques such as adsorption, entrapment, and electrostatic interaction [18]. The immobilization of enzymes has become a rapidly expanding area of study due to its advantages in recovering and reusing expensive enzymes, simplifying enzyme separation from catalytic products, and improving enzyme stability during storage and operational processes [19].

Tannic acid (TA) is a naturally occurring, water-soluble polyphenolic compound that has attracted considerable interest in biomedical and biotechnological fields due to its ability to form multiple interactions with biomolecules. Its numerous hydroxyl groups and quinone structures facilitate strong hydrogen bonding and covalent interactions with proteins, carbohydrates, and other nucleophilic species [20, 21]. These features also enable TA to self-polymerize into poly (tannic acid) coatings on various support surfaces [22–26]. Such coatings support enzyme immobilization via Schiff base reactions and/or Michael addition with nucleophilic groups such as amines and thiols [21, 24, 27]. This ability to form stable covalent linkages makes TA a versatile natural linker that can enhance enzyme loading, stability, and functional performance on support materials [22].

Chia seed gum, present in the coat, becomes easily visible and extractable when exposed to water. Chia gum is an anionic heteropolysaccharide composed of 4-O-methyl-𝛼-D-glucopyranosyluronic acid units, 𝛽-D-xylopyranosyl, and 𝛼-D-glucopyranosyl. It exhibits a greater ability to absorb, retain, and bind water [28]. Due to its hydrophilic characteristics, it can trap significant amounts of water between its chains, preventing water loss from the encapsulating support and inhibiting enzyme leaching. Furthermore, proteins are positively charged, while gums are negatively charged. The interaction between these polymers, resulting from their opposing charges, leads to complex coacervation. Coacervation has a wide range of applications, including encapsulating polyunsaturated oils and other bioactive food ingredients, stabilizing emulsions, and delivering bioactive substances in microcapsules [29, 30].

In this study, tannic acid (TA) was used as a natural linker molecule to immobilize HRP on chia gum (CG) as natural support for enhancing its mechanical properties of enzyme for the first time. However, both CG and TA are low-cost, natural, and biodegradable materials making the system economically and environmentally attractive. The characteristics of immobilized HRP, such as environmental tolerance, catalytic activity, and reusability were studied. Additionally, a potential industrial application for azo dye removal was also explored.

## Materials and methods

### Materials

Horseradish peroxidase (HRP) was previously purified by Mohamed et al. [31] with specific activity of peroxidase 7961uints/mg protein. Chia seeds were purchased from the local market in Cairo, Egypt.

### Chia gum preparation

Chia seeds were soaked in distilled water at a ratio of 1:30 for 1 h at 40 °C. Filtration was used to separate the gum, and one volume of gum was mixed with three volumes of ethanol. The precipitate was collected, dried, and crushed.

### Measuring peroxidase activity

According to Miranda et al. [32], the peroxidase activity was assessed with 20 mM sodium acetate buffer, pH 5.5, 8 mM H_2_O_2_, 40 mM guaiacol, and HRP enzyme present. One unit of enzyme is the change in absorbance (A470) of 1.0/min. The Activity of perxidase was calculated using the following equation:

$$\:\text{Activity}\:\text{of}\:\text{perxidase}=\Delta\text{A}/\Delta\text{t}$$

Where ΔA is the change in absorbance, Δt is the change in time.

### Preparation of support

The following is a description of how tannic acid (TA) activates chia gum (CG): For one hour, 30 mL of TA (0.9 mg) and 30 mL of the CG solution (150 mg) were mixed in distilled water and stirred at 1000 rpm at room temperature. Absolute ethanol was added to the solution in a 1:3 (v/v) ratio. Before the immobilization procedure, the precipitated (CG-TA) was dried, powdered, and kept at 4 °C.

### Immobilization process

To immobilize the horseradish peroxidase (HRP), 1 milliliter of enzyme containing 15 units was combined with CG-TA. The mixture was gently vortexed for two minutes and allowed to react at room temperature for one hour. After five hours of freezing at -80 °C to preserve structure, the CG-TA-HRP mixture was lyophilized overnight at -50 °C. The immobilization efficiency percentage was calculated using the following formula.

$$\begin{aligned} & immobilization\,\,efficiency\left( \% \right)\\&= \frac{\begin{gathered} Activity\,\,of\,\,immobilized\, \hfill \,enzyme \hfill \end{gathered} }{\begin{gathered} initial\,\,activity\,\,of\,\,soluble\, \hfill \,enzyme \hfill \end{gathered} } \\ & \times 100 \\ \end{aligned} $$

### Surface morphology

The surface morphology of supports (CG-TA) and the immobilized enzyme (CG-TA-HRP) were examined using an energy dispersive X-ray (EDX) fitted with a Holland Field Emission Scanning Electron Microscope (FE-SEM, Quanta FEG250) at an accelerating voltage of 20 kV.

### Fourier transform infrared analysis (FTIR analysis)

CG-TA and CG-TA-HRP spectra were obtained using the FTIR Spectrometer (Bruker ALPHA-FTIR-Spectrometer). The wavelength range for platinum-attenuated reflection was 400–4000 cm^1^.

### Thermal properties

The thermal properties of the prepared CG-TA and CG-TA-HRP were examined using thermogravimetric analyser (TGA) and differential scanning calorimeter (DSC). Sample runs were carried out between 40 and 600 °C at a steady heating rate of 10 °C/min.

### Reusability of immobilized enzyme

Following a thorough enzyme assay, the CG-TA-HRP was taken out of the reaction mixture, cleaned with buffer, and the immobilized enzyme’s reusability was determined. After that, it was used once again in the same reaction mixture.

### pH optimum

Both soluble HRP and CG-TA-HPR optimal pH’s were established in different pH ranges (4.0–8.0), and the residual activity was assessed using conventional assay procedures.

### Temperature optimum and stability

The optimal temperature for both soluble-HRP and CG-TA-HPR at different temperatures (20–70 °C) was evaluated using the standard test. Before adding substrate and cooling in an ice bath, the CG-TA-HPR or the soluble-HRP was incubated for an hour at different temperatures (30–70 °C) in the temperature stability assay. The residual activity was then evaluated under standard assay conditions.

### Determination of Km and Vmax values

Lineweaver-Burk plots were used to determine the Km and Vmax values of CG-TA-HPR and soluble-HRP using H_2_O_2_ or guaiacol at varying doses.

### Effect of metals and certain compounds

The activity of soluble HRP and CG-TA-HPR was determined under normal assay conditions, as well as the impact of certain metal ions (Mg^2+^, Ca^2+^, Al^3+^, Cu^2+^, Ni^2+^, Zn^2+^, and Hg^2+^) and denaturation chemicals (Urea, Triton X-100, isopropanol, and dimethyl-sulfoxide).

### Methyl orange decolorization

The reaction mixture consisted of 20 units of soluble HRP or CG-TA-HRP, 0.05 M sodium acetate buffer (pH 6.0), 0.05 M methyl orange, and 0.008 M H_2_O_2_ in a total volume of 6.0 ml. A 1.0 ml sample was taken every hour while the mixture was shaken at 100 rpm. The absorbance was then measured at 465 nm.

$$\:Decolorization\left(\%\right)=\left[\frac{\left(A0-At\right)}{A0}\right]\times\:100$$

The methyl orange absorbance is denoted by A0, while the treated methyl orange absorbance is represented by At.

All experimental procedures were carried out in compliance with relevant guidelines.

## Results and discussion

### Immobilization of HRP on CG-TA

The strategies of the immobilization process are based on TA-mediated surface modification of CG and HRP. TA was first polymerized and coated onto the surface of CG, and the formed polymer can be used as a reactive surface coating for HRP immobilization via the Schiff base formation and/or the Michael addition reaction between quinone groups on poly (tannic acid) layer surfaces and exposed amine groups on HRP. Moreover, CG can trap significant amounts of water between its chains, preventing water loss from the gum and inhibiting enzyme leaching.

The immobilization of HRP was investigated at various pH values (5.0, 7.0, and 8.0) and TA concentrations (0.3, 0.6, and 0.9 mg) (Table 1). The experiment used 150 mg of CG and 20 units of HRP. At pH 7.0 and 0.9 mg TA, the highest immobilization efficiency (80%) was achieved. At pH 8.0 and 0.3 mg TA, the lowest immobilization efficiency (33%) was noted. A low concentration of TA as a crosslinker may be the cause of this decrease in immobilization efficiency.

**Table 1:** Impact of pH and Tannic acid concentrations on HRP immobilization proficiency at 150 mg of chia gum

Tannic acid (mg)	Immobilization efficiency %		
	pH 5.0	pH 7.0	pH 8.0
0.3	44 ± 1.2	56 ± 22	33 ± 1.1
0.6	55 ± 2.4	68 ± 2.8	42 ± 1.5
0.9	66 ± 3.9	80 ± 3.3	52 ± 2.1

**Fig. 1:**
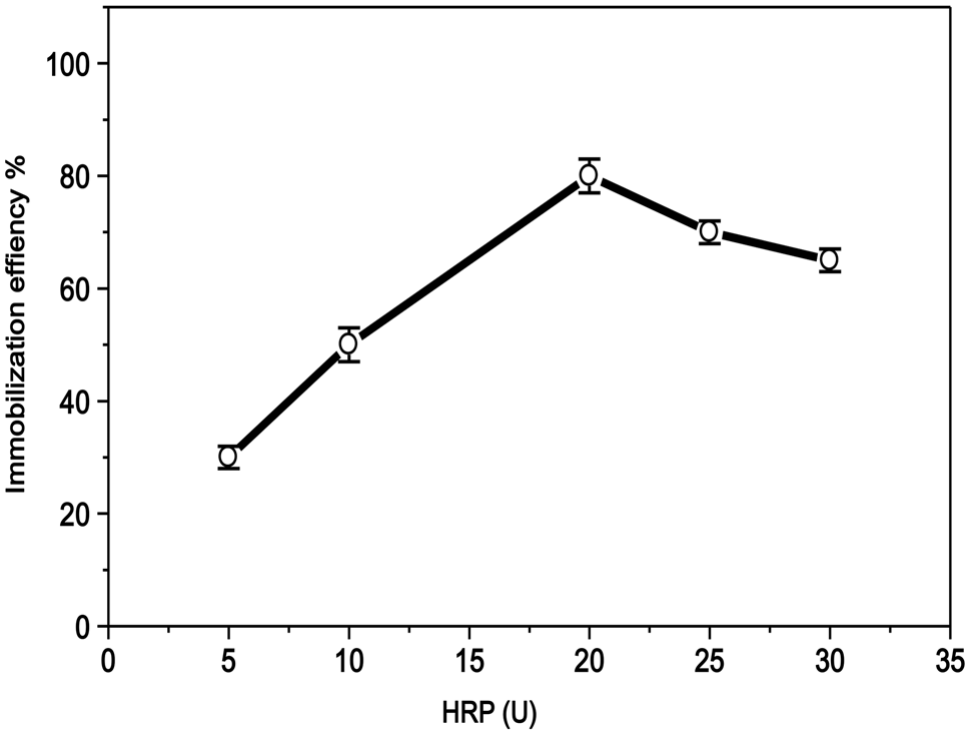
Effect of HRP at different concentrations (5–30 U) on the immobilization efficiency

The impact of different HRP concentrations (5–30 units) on the immobilization efficiency percentage under the ideal immobilization circumstances (pH 7.0 and 0.9 mg TA), as determined previously, is depicted in Fig. 1. At 20 units, the highest immobilization efficiency of 80% was observed. The immobilization efficiency (63%) decreased somewhat when the enzyme concentration was increased to 30 units. This decrease could result from an excessive buildup of the enzyme on the polymer surface, which would decrease the diffusion of the substrate [33].

### Surface-morphology characterization

**Fig. 2:**
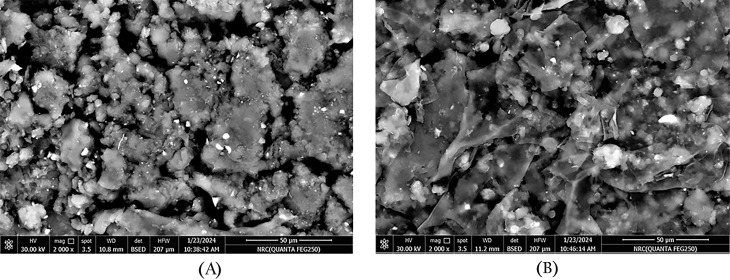
SEM micro-images of the prepared CG-TA support (**A**) and immobilized CG-TA-HRP (**B**)

The surface morphology of the immobilized CG-TA-HRP and the CG-TA support was examined using SEM micro-images. Figure 2A shows that the CG-TA is a micropore-free, sawdust-like material. The immobilized HRP appeared as small, irregular particles on the CG-TA surface, indicating that TA crosslinked HRP to CG (Fig. 2B). This close contact between the HRP and the prepared CG-TA support indicates robust immobilization through mechanisms such as covalent bonding or physical adsorption. Changes in the immobilized HRP’s coating and surface morphology on the chia gum alginate were previously documented [34].

**Fig. 3:**
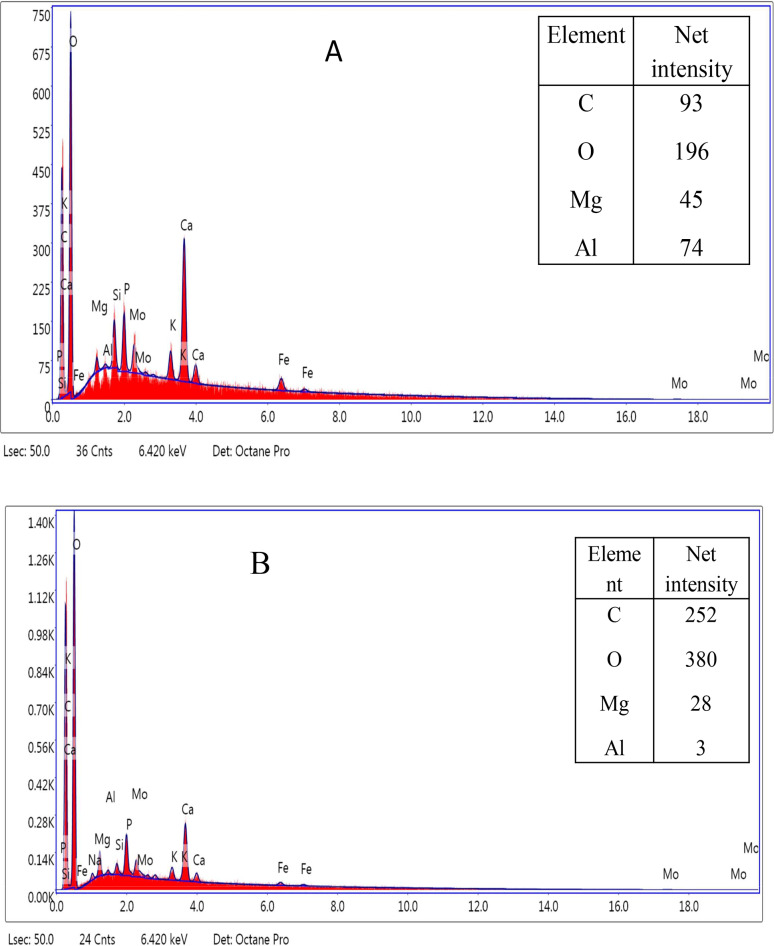
EDX of the prepared CG-TA support (**A**) and immobilized CG-TA-HRP (**B**)

Both CG-TA and CG-TA-HRP were examined using EDX to identify the elements that were present on the surface. The existence of several elements in both samples, including carbon C, oxygen O, aluminum Al, and magnesium Mg, is shown by the EDX results in Fig. 3. According to the results, the CG-TA-HRP had a greater net oxygen content intensity (380) than the CG-TA (196). Therefore, the effective immobilization of HRP on CG-TA was validated by the enhanced oxygen content intensity. The immobilization of HRP onto an iron magnetic nanocomposite has shown similar behavior [35]. Additionally, Sahare et al. [36] noted that the immobilization of HRP onto mesoporous silicon/silica micro-particles required a higher oxygen content.

### FTIR-analysis

**Fig. 4:**
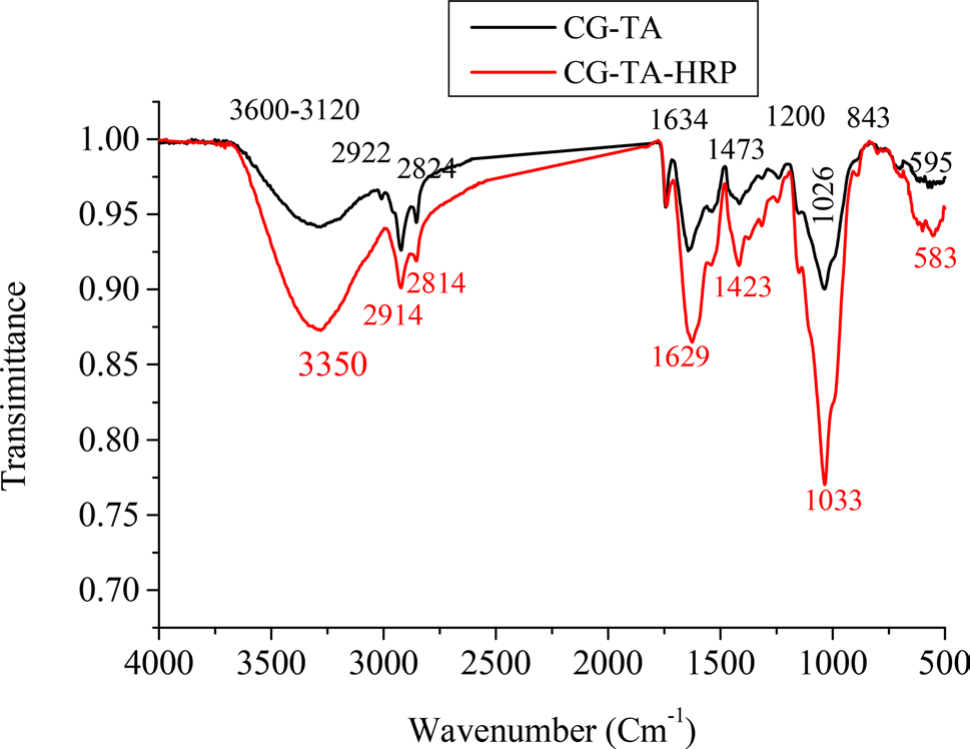
FTIR spectra of the prepared CG-TA support and immobilized CG-TA-HRP

As shown in Fig. 3, the FTIR spectrum of CG-TA exhibits peaks at 3500–3100 cm^−^¹ (O–H stretching), 2922 cm^−^¹ and 2824 cm^−^¹ (symmetric and asymmetric C–H_2_ stretching), and 1634 cm^−^¹ and 1473 cm^−^¹ (asymmetric and symmetric COO⁻ vibrations from uronic acids and TA) [37]. Additional peaks appear at 1200 cm^−^¹ (C–O stretching) and 843 cm^−^¹ (C–C and C–O–C vibrations) [34]. After HRP immobilization, the FTIR spectrum of CG-TA-HRP (Fig. 4) shows a more defined band at 3350 cm^−^¹, due to the overlapping of O–H and amide A (N–H stretching). New peaks appear at 1629 cm^−^¹ (amide I, C = O stretching), 1423 cm^−^¹ (amide II, N–H deformation), and 1033 cm^−^¹ (amide III, C–N stretching and N–H bending). A peak at 583 cm^−^¹ corresponds to C–H bending. These spectral changes confirm successful HRP immobilization on the CG-TA matrix and indicate an amination reaction [38].

### Thermal properties

**Fig. 5:**
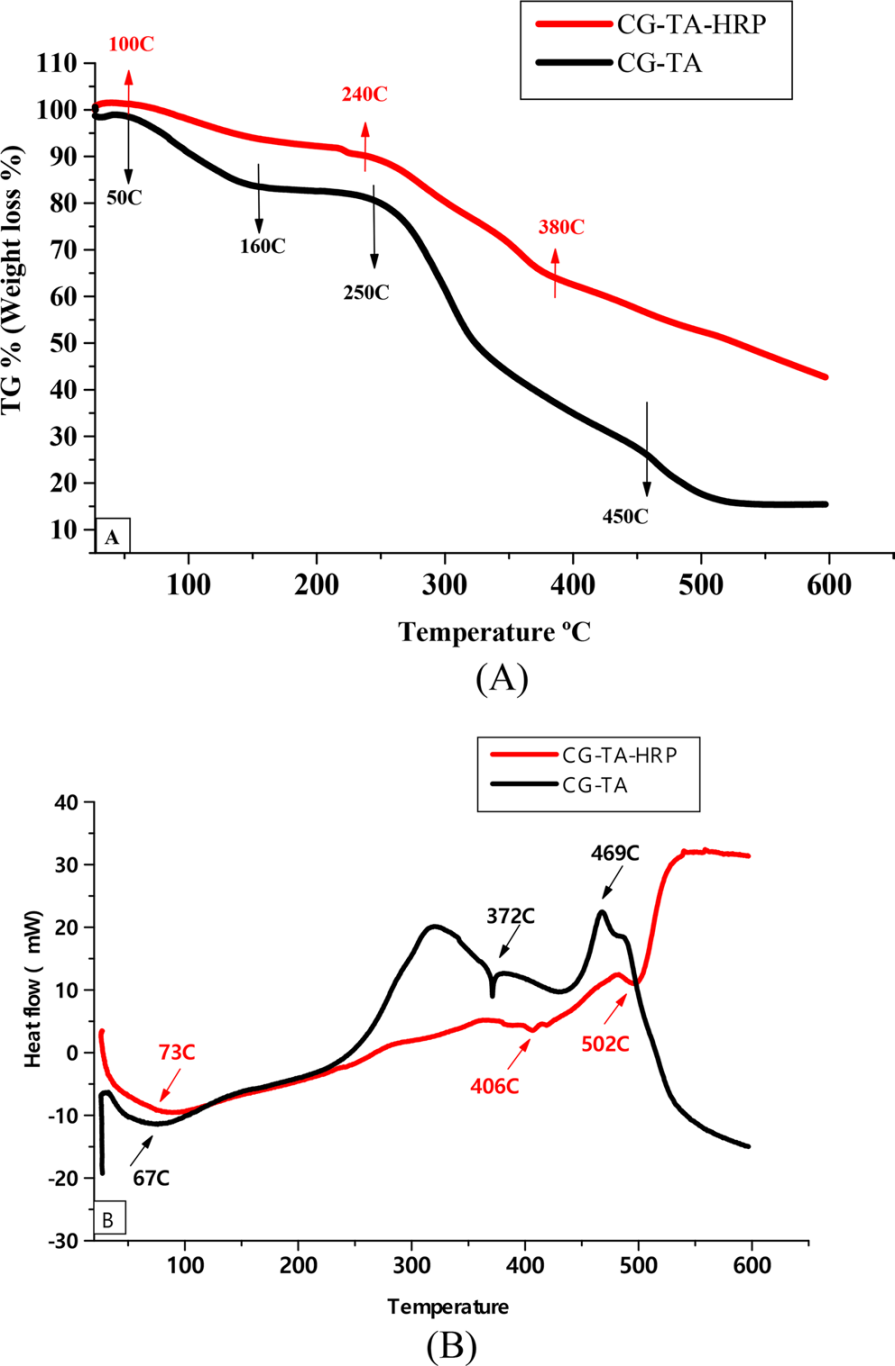
(**A**) TGA and (**B**) DSC curves of the CG-TA and CG-TA-HRP

TGA and DSC experiments were used to analyze the CG-TA and CG-TA-HRP’s thermal characteristics. As shown in Fig. 5A, the TGA analysis revealed that the initial thermal deterioration for CG-TA occurred between 50 and 160 °C with a mass loss of 17%, and the initial thermal degradation for CG-TA-HRP occurred between 100 and 240 °C with a mass loss of 10%. This initial mass loss is associated with the evaporation of the samples’ moisture content or water content. In addition, the second mass breakdown was detected between 250 and 450 °C for CG-TA and 240–380 °C for CG-TA-HRP with 75 and 35% mass lose, respectively. CG-TA and CG-TA-HRP both maintained 15 and 43% of their masses at 600 °C. The similar results of TGA were detected by encapsulation of phenolic compounds in chia gum [39]. By monitoring the energy transfer, differential scanning calorimetry (DSC) can identify any changes in the materials’ physicochemical characteristics [40]. DSC analyzed the CG-TA and CG-TA-HRP to verify the physical condition and interaction of the HRP in the CG-TA. Figure 5B presented the findings. The CG-TA DSC spectrum displays a broad endothermic peak at around 67 °C (between 40 °C and 100 °C), which could be related to the water content becoming dehydrated. Following HRP immobilization, this peak was moved to 73 °C (CG-TA-HRP). Two CG-TA peaks at 372 °C and 469 °C were shifted to CG-TA-HRP peaks 406 °C and 502 °C. The relationship between HRP and CG-TA is supported by these modifications. These findings are comparable to those of the chia gum DSC [41]. The TGA and DSC results suggest that CG-TA and CG-TA-HRP have outstanding thermal stability, which opens the possibility of using them in high-temperature operations.

### Biochemical characterization

**Fig. 6:**
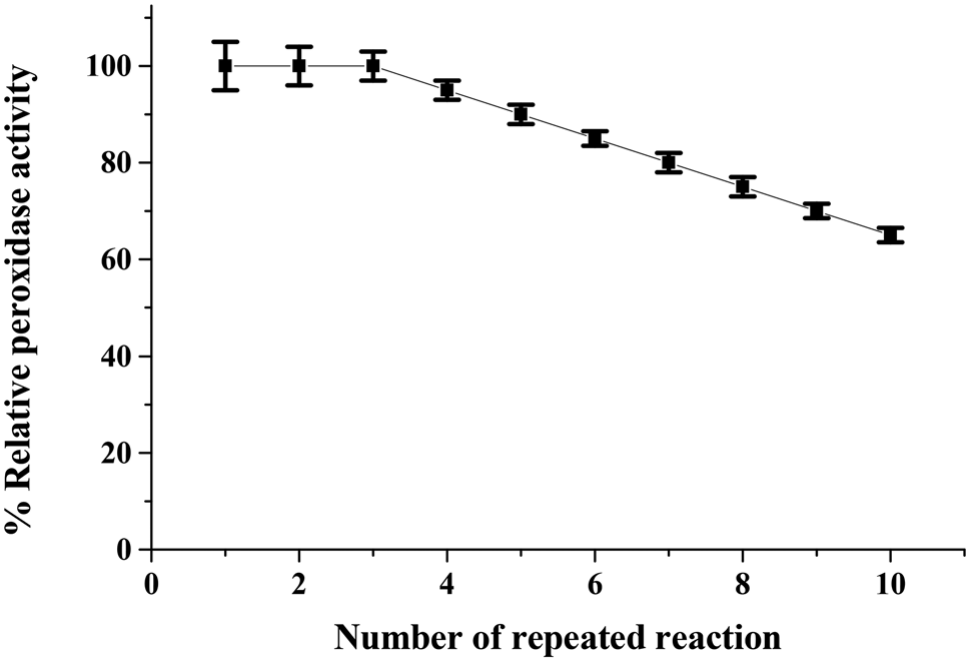
Number of reuses of the CG-TA-HRP

The operational stability of the immobilized HRP was evaluated through repeated catalytic cycles (Fig. 6). The CG-TA-HRP system retained approximately 65% of its initial activity after ten cycles, indicating strong enzyme–support interactions and minimal leaching. This improved reusability confirms the enhanced structural integrity and durability of the enzyme in its immobilized form, making it suitable for practical applications requiring sustained activity. A little decline in activity during reuse may be attributed to enzyme inhibition by H_2_O_2_ and/or the gradual degradation of the CG-TA support material, which can affect enzyme binding and structural integrity [42]. In comparison to cashew gum-HRP, which retained 50% of its initial activity after nine reuses, this result is more favorable [43]. After six repeats, 65.8% of the original activity was preserved by the chitosan-HRP [44].

**Fig. 7:**
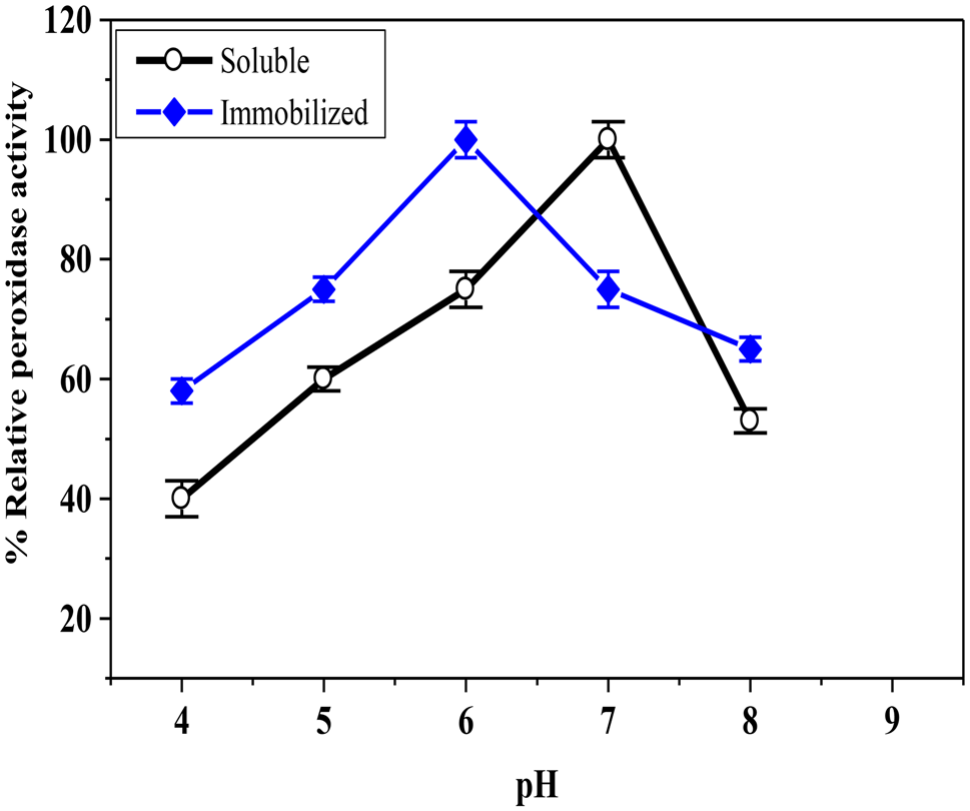
pH optima for soluble HRP and CG-TA-HRP

The pH-optimal activity of soluble HRP and CG-TA-HRP is displayed in Fig. 7. The pH optimum of the soluble HRP was 7.0, whereas the pH of CG-TA-HRP was 6.0. However, CG-TA-HRP retained a higher activity than soluble-HRP at lower and higher pH’s, demonstrating that the immobilized enzyme was less responsive to pH variations than the soluble enzyme. This improved stability is likely due to the protective effect of the CG-TA matrix, which helps maintain enzyme structure under pH stress. A broad acidic pH optimum was observed for Concanavalin A-HRP (pH 4.0 to 5.0) [45]. On the other hand, the pH of the immobilized enzyme on acrylic polymer was changed from 7.0 for soluble HRP to 7.5–8.0 [46]. Additionally, the pH was adjusted for the immobilized-HRP on nanodiamond from 7.0 for soluble-HRP to 7.5 [47]. These differences highlight how the chemical properties of each support material affect the enzyme’s response to pH. The pH stability of CG-TA-HRP indicates a unique and favorable interaction between the enzyme and the CG-TA matrix, making it well-suited for use in environments with changing pH conditions.

**Fig. 8:**
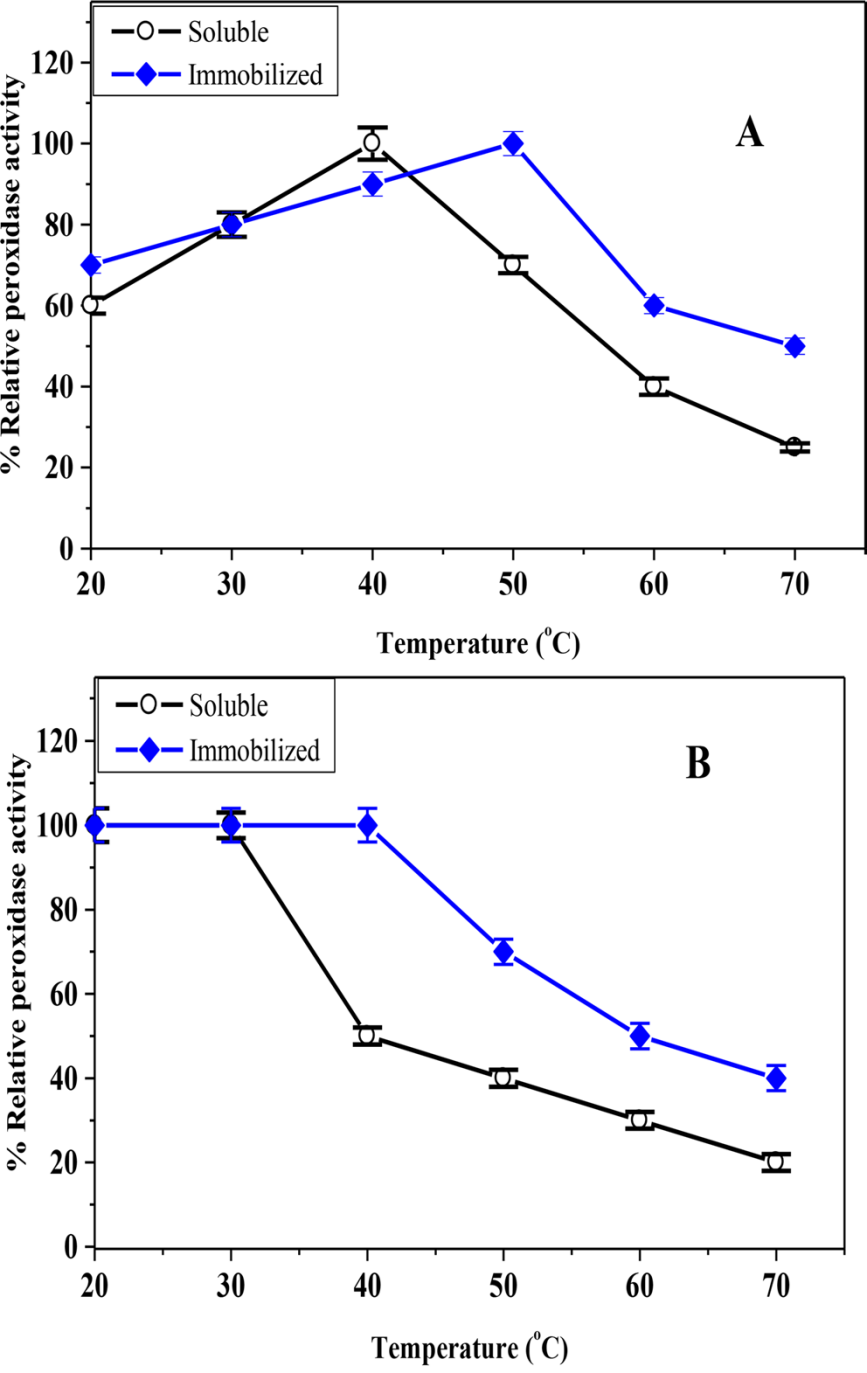
Temperature-optimum (**A**) and thermal-stability (**B**) of the soluble HRP and CG-TA-HRP

The optimum temperature for the soluble-HRP was 40 °C, as opposed to 50 °C for the CG-TA-HRP that was prepared (Fig. 8A). This shift suggests that immobilization on the CG-TA matrix enhances the enzyme’s thermal tolerance, likely due to the structural rigidity and protective structure provided by the support. Such stabilization helps reduce enzyme unfolding or denaturation at elevated temperatures. For chitosan-HRP, a similar optimum temperature of 45 °C was previously found [44]. Furthermore, the thermal stability of soluble-HRP and CG-TA-HRP was evaluated (Fig. 8B). After one hour of incubation, the soluble HRP and CG-TA-HRP remained stable at temperatures as high as 30 °C and 40 °C, respectively. This improved thermal resilience is consistent with findings from HRP immobilized on chitosan-coated woven polyester fabric, where the immobilized form showed greater resistance to thermal deactivation [48]. These results demonstrate that CG-TA immobilization improves enzyme robustness under thermal stress, which is advantageous for practical applications in varying temperature conditions.

**Fig. 9:**
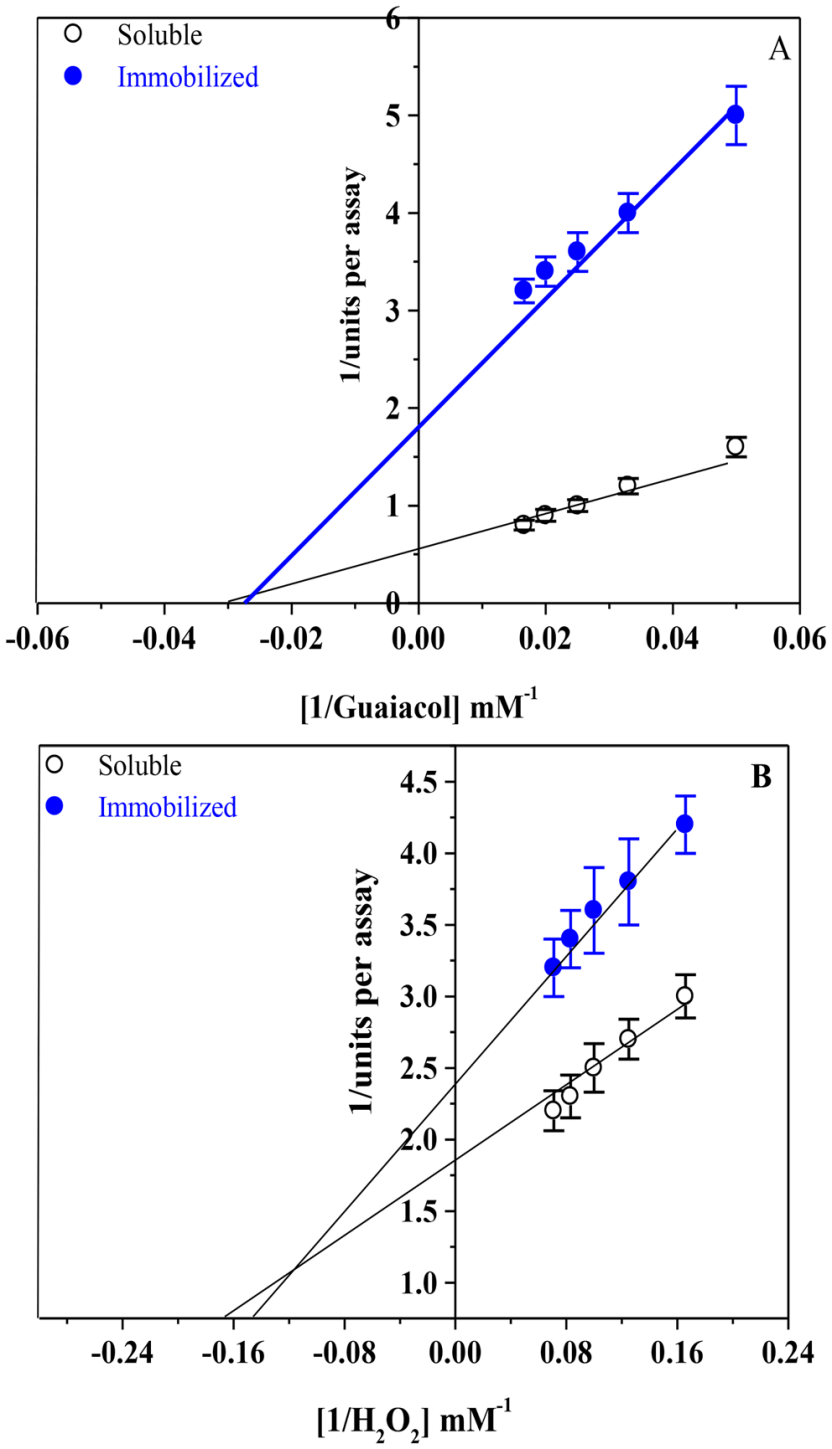
Lineweaver-Burk plots of soluble HRP and CG-TA-HRP reaction velocities to guaiacol (**A**) and H_2_O_2_ (**B**) concentrations

The immobilization procedure typically affects the diffusion of the substrate toward the enzyme’s active site, which in turn influences the value of Km [35]. The Km values for H_2_O_2_ and guaiacol were 6.0 and 6.9 mM for soluble HRP, and 33 and 37 mM for CG-TA-HRP, respectively (Fig. 9a, b). These results indicate that soluble HRP has a higher affinity for both substrates compared to CG-TA-HRP. The increased Km values after immobilization suggest a reduction in substrate accessibility or binding efficiency. This change could be attributed to steric hindrance or diffusion limitations caused by the CG-TA matrix, which may partially block or alter the enzyme’s active site. Additionally, conformational changes in the enzyme upon attachment to the support may alter its binding behavior [49, 50]. Following conjugation with the support, the enzyme’s structure may be altered, impacting both the substrate entry and the geometry of the catalytic site [51, 52]. Similar observations have been reported for other immobilized systems, such as HRP immobilized on iron magnetic nanoparticles showed higher Km values compared to its soluble form [35], indicating reduced substrate affinity. However, in contrast, HRP immobilized on chitosan-coated nonwoven polyester fabric exhibited a lower Km than soluble HRP [48], which highlights how the choice of support and immobilization method can significantly influence enzyme kinetics.

Table 2 shows the oxidizing affinities of soluble HRP and CG-TA-HRP for various substrates, including *p*-aminoantipyrine, pyrogallol, *o*-dianisidine, *o*-phenylenediamine, and guaiacol. The CG-TA-HRP demonstrated superior efficiency for each substrate examined, in contrast to soluble-HRP. This enhanced activity suggests that immobilization on the CG-TA matrix may have improved the orientation or accessibility of the active site, promoting more efficient substrate conversion. Immobilization also improves enzyme activity by stabilizing its structure and making it easier for substrates to reach the active site. A previous evaluation demonstrated that HRP immobilized on iron magnetic nanoparticles showed a higher relative activity in oxidizing the same substrates compared to soluble HRP [35], supporting the concept that immobilization can enhance enzyme efficiency depending on the support material and interaction strength.

**Table 2:** Substrate specificity of the soluble HRP and CG-TA-HRP

Substrate	Relative peroxidase activity%	
	Soluble-HRP	TA-CG-HRP
Guaiacol	100 ± 4.1^a^	100 ± 4.0^a^
*o*-Dianisidine	90 ± 3.4^b^	130 ± 6.2^b^
*o*-Phenylenediamine	50 ± 2.0^b^	102 ± 104.2 ^b^
Pyrogallol	42 ± 1.8^b^	62 ± 1.7 ^b^
*p*-Aminoantipyrine	20 ± 1.0^b^	50 ± 2.2^b^

Values with different superscripts (a, b) were significantly different at (*P* < 0.01)

Since wastewater contains significant amounts of heavy metals, the effect of different metal ions on the produced CG-TA-HRP was studied. Compared to soluble-HRP, the CG-TA-HRP exhibited enhanced resistance to heavy metals. The majority of metal ions tested exhibited a partial inhibition of soluble HRP compared to CG-TA-HRP (Table 3). CG-TA-HRP exhibited a lower level of inhibition by Hg² (44%) than soluble HRP (21%). Despite the presence of heavy metals, the stability of CG-TA-HRP against various metal ions suggests its potential as an immobilized enzyme for remediating aromatic contaminants. These findings demonstrate that the immobilization of HRP on the CG-TA matrix greatly improves its operational stability under harsh conditions, including exposure to toxic metal ions. This enhanced tolerance is likely due to the protective microenvironment formed by the CG-TA network, which shields the enzyme and limits direct contact between metal ions and the active sites of HRP. As a result, the CG-TA-HRP system remains functionally active even in contaminated environments, highlighting its potential as a stable and reusable biocatalyst for the treatment of aromatic pollutants in wastewater. Numerous investigations revealed that the immobilized HRP was more resistant to heavy metals than the soluble enzyme [53, 54].

**Table 3:** Effect of 10 mM metal ions on the soluble HRP and CG-TA-HRP

Metals	Relative peroxidase activity%	
	Soluble-HRP	CG-TA-HRP
Cu^2+^	69 ± 3.3^b^	100 ± 6.2 ^a^
Ni^2+^	66 ± 2.0^b^	100 ± 4.5^b^
Zn^2+^	52 ± 2.1^b^	80 ± 3.2 ^b^
Mg^2+^	60 ± 3.2^b^	75 ± 4.5 ^b^
Ca^2+^	44 ± 1.6^a^	62 ± 2.5 ^b^
Al^3+^	33 ± 1.3^b^	50 ± 1.2 ^a^
Hg^2+^	21 ± 1.1^b^	44 ± 1.8 ^a^

Values with different superscripts (a, b) were significantly different at (*P* < 0.01)

Protein unfolding was identified through interaction with a high urea concentration [55]. When soluble-HRP was exposed to 4.0 M urea, 23% of its activity was preserved, but CG-TA-HRP retained 44% of its activity (Table 4). These observations confirmed CG-TA-HRP’s robust resistance to urea. Similar to this, chitosan-HRP and Concanavalin A-pointed gourd peroxidase were resistant to urea [48, 45]. To remove dye pollutants from wastewater, it is essential to test the stability of HRP in the presence of detergents. When compared to soluble-HRP, CG-TA-HRP demonstrated significantly greater stability against Triton X-100 (Table 4). Several studies have shown that immobilized peroxidases are stable in the presence of Triton X-100 [48, 45]. The stability of HRP against organic solvents needs to be assessed, as wastewater contains various organic solvents. In the presence of 5% and 10% isopropanol, CG-TA-HRP exhibited significantly greater stability than soluble-HRP, retaining 60%, 52%, and 45%, 33% of its initial activity, respectively (Table 4). Both chitosan-HRP and wool-HRP also exhibited resistance to organic solvents [46, 53]. This enhanced tolerance is attributed to the CG-TA matrix, which offers a protective framework that stabilizes the enzyme’s structure and reduces direct exposure to harsh chemicals. Such stability is essential for maintaining catalytic performance in real-world wastewater treatment applications.

**Table 4:** Effect of urea, triton X-100, and isopropanol on the soluble HRP and CG-TA-HRP

Substrate	Concentration	Relative peroxidase activity%	
		Soluble-HRP	TA-CG-HRP
None	----	100 ± 5.3^a^	100 ± 4.2^a^
Urea	2 M	36 ± 1.8^b^	52 ± 2.2^b^
	4 M	23 ± 1.8^b^	42 ± 2.2^b^
Triton X-100	5%	28 ± 2.0^b^	55 ± 1.5 ^b^
	10%	18 ± 2.0^b^	33 ± 1.5 ^b^
Isopropanol	5%	45 ± 1.7^b^	60 ± 4.7 ^b^
	10%	33 ± 1.7^b^	52 ± 4.7 ^b^

Values with different superscripts (a, b) were significantly different at (*P* < 0.01)

**Fig. 10:**
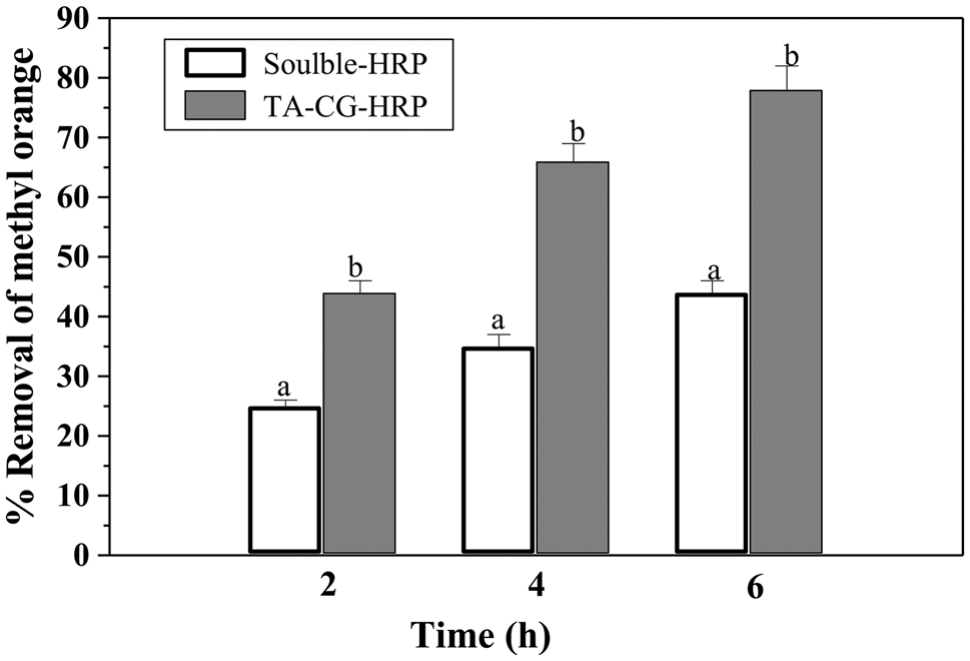
Influence of soluble HRP and CG-TA-HRP on methyl orange elimination

Methyl orange, an azo dye, was decolorized using both soluble and immobilized forms of HRP (Fig. 10). The immobilized enzyme demonstrated better performance than the soluble one in removing methyl orange. After six hours of incubation, 78% of the methyl orange was decolorized in the immobilized form, while only 44% was removed in the soluble form. However, the soluble peroxidases from horseradish and *Ficus sycomorus* latex removed the methyl orange by 60% and 66%, respectively [4]. During the 5-hour incubation period, soluble HRP removed 45% of the methyl orange, whereas immobilized HRP decolorized 82% of the dye [56]. Recent work using chitosan-coated magnesium manganese oxide (CH-MMO) for HRP immobilization showed enhanced dye degradation and enzyme stability [57]. Similarly, our CG-TA matrix achieved strong stability and activity, but with the added benefit of being entirely natural and eco-friendly. This emphasizes that natural biopolymers (CG-TA) can be effective, sustainable alternatives to synthetic hybrid supports.

## Conclusion

In this study, horseradish peroxidase (HRP) was successfully immobilized on a newly developed chia gum–tannic acid (CG-TA) support, where chia gum served as the matrix and tannic acid acted as a natural cross-linker. The resulting CG-TA-HRP system demonstrated high immobilization efficiency, strong reusability, and significantly enhanced stability under various denaturing conditions, including heat, pH extremes, heavy metals, detergents, organic solvents, and urea. It also exhibited superior performance in azo dye removal compared to soluble HRP. The use of biodegradable, non-toxic, and inexpensive materials—chia gum and tannic acid—makes the approach not only eco-friendly but also economically viable for large-scale applications. This work presents a newly developed CG-TA matrix for HRP immobilization, offering a simple, sustainable, and cost-effective platform for industrial and environmental biocatalysis.

## Data Availability

All data generated and analyzed in this study are included in this published article.
